# Collapsin Response Mediator Protein 4 (CRMP4) Facilitates Wallerian Degeneration and Axon Regeneration following Sciatic Nerve Injury

**DOI:** 10.1523/ENEURO.0479-19.2020

**Published:** 2020-03-02

**Authors:** Marie-Pier Girouard, Tristan Simas, Luyang Hua, Barbara Morquette, Mohamad R. Khazaei, Nicolas Unsain, Aaron D. Johnstone, Isabel Rambaldi, Ricardo L. Sanz, Marie-Eve Di Raddo, Kanchana K. Gamage, Yu Yong, Dianna E. Willis, Valerie M. K. Verge, Philip A. Barker, Christopher Deppmann, Alyson E. Fournier

**Affiliations:** 1 Department of Neurology and Neurosurgery, Montréal Neurological Institute and Hospital, Montréal, Québec H3A 2B4, Canada; 2 Instituto de Investigación Médica Mercedes y Martín Ferreyra (INIMEC), Consejo Nacional de Investigaciones Científicas y Técnicas (CONICET), 5016 Córdoba, Argentina; 3 McGill University, Montréal, Québec H3A 0G4, Canada; 4 Department of Biology, University of Virginia, Charlottesville, Virginia 22903; 5 Brain and Mind Research Institute, Weill Cornell Medicine, New York, New York 10065; 6 Burke Institute, Weill Cornell Medicine, White Plains, New York 10605; 7 Department of Anatomy, Physiology and Pharmacology, University of Saskatchewan-CMSNRC, Saskatoon, Saskatchewan S7N 5E5, Canada; 8 Department of Biology, University of British Columbia, Kelowna, British Columbia V1V 1V7, Canada

**Keywords:** collapsin response mediator protein, Wallerian degeneration, neuronal regeneration, calpain, sciatic nerve injury, sensory neurons

## Abstract

In contrast to neurons in the CNS, damaged neurons from the peripheral nervous system (PNS) regenerate, but this process can be slow and imperfect. Successful regeneration is orchestrated by cytoskeletal reorganization at the tip of the proximal axon segment and cytoskeletal disassembly of the distal segment. Collapsin response mediator protein 4 (CRMP4) is a cytosolic phospho-protein that regulates the actin and microtubule cytoskeleton. During development, CRMP4 promotes growth cone formation and dendrite development. Paradoxically, in the adult CNS, CRMP4 impedes axon regeneration. Here, we investigated the involvement of CRMP4 in peripheral nerve injury in male and female *Crmp4^−/−^* mice following sciatic nerve injury. We find that sensory axon regeneration and Wallerian degeneration are impaired in *Crmp4^−/−^* mice following sciatic nerve injury. *In vitro* analysis of dissociated dorsal root ganglion (DRG) neurons from *Crmp4^−/−^* mice revealed that CRMP4 functions in the proximal axon segment to promote the regrowth of severed DRG neurons and in the distal axon segment where it facilitates Wallerian degeneration through calpain-dependent formation of harmful CRMP4 fragments. These findings reveal an interesting dual role for CRMP4 in proximal and distal axon segments of injured sensory neurons that coordinately facilitate PNS axon regeneration.

## Significance Statement

Peripheral nervous system (PNS) neurons spontaneously regenerate after injury; however, functional deficits often arise as a result of slow or misguided repair. Regrowth of the proximal axon segment coordinated with efficient Wallerian degeneration is important for optimal recovery. CRMP4 is a cytoskeletal regulatory protein with growth-promoting functions in the developing nervous system and growth-inhibitory roles in damaged adult CNS neurons. Here, we identify a proregenerative role for CRMP4 in peripheral nerve regeneration through the coordinated regulation of both axon regrowth and Wallerian degeneration.

## Introduction

Peripheral nervous system (PNS) and CNS neurons respond differently to injury because of distinct extracellular environments at the lesion site and differences in intrinsic signaling. Regeneration of axotomized (PNS) neurons is supported by the expression of regeneration-associated genes (RAGs) and a growth-permissive environment, whereas injured CNS neurons fail to re-express RAGs and encounter glial-derived inhibitors that impede regeneration (Huebner and Strittmatter, 2009; Mokarram and Bellamkonda, 2011; Mietto et al., 2015). Even in the PNS, long-distance regeneration is slow and recovery is often incomplete, resulting in the partial or complete loss of sensory and/or motor functions (Mokarram and Bellamkonda, 2011). A stereotypical sequence of events is initiated on PNS injury. The proximal axon segment reforms a growth cone that drives axon extension and regeneration, while the detached distal axon segment undergoes Wallerian degeneration and subsequent fragmentation (Waller, 1850; Erez and Spira, 2008; Kamber et al., 2009; Ghosh-Roy et al., 2010; Bradke et al., 2012). Phagocytosis of cellular debris further contributes to a growth-permissive environment (Bruck, 1997; Lewis and Kucenas, 2014; Brosius Lutz et al., 2017). Impaired regeneration of injured sensory and motor neurons in slow degenerating *Wld^s^* mice suggests that optimal regeneration requires proper coordination of proximal axon repair and distal axon degeneration (Bisby and Chen, 1990; Brown et al., 1992, 1994). Thus, proteins regulating both processes could represent therapeutic targets for promoting recovery following PNS injury.

Collapsin response mediator proteins (CRMPs) are a family of cytosolic phospho-proteins that regulate cytoskeletal dynamics during development and after injury (Alabed et al., 2007; Khazaei et al., 2014; Nagai et al., 2015,  2016; Tan et al., 2015). The CRMP4 family member has two splice isoforms, referred to as the long isoform of CRMP4 (CRMP4L) and the short isoform of CRMP4 (CRMP4S), and is an important neurodevelopmental molecule promoting axonal extension and dendrite branching (Quinn et al., 2003; Niisato et al., 2012; Khazaei et al., 2014; Tan et al., 2015; Cha et al., 2016). However, in the adult CNS, CRMP4-null mice exhibit enhanced neuronal regeneration and reduced inflammation following spinal cord injury, suggesting that CRMP4 impedes regeneration in this context (Nagai et al., 2015, 2016). The inhibitory role of CRMP4 is partly due to its function in transducing signals from myelin-associated inhibitors (MAIs) and chondroitin sulfate proteoglycans (CSPGs; Alabed et al., 2007). In the adult mammalian PNS, CRMP4 is upregulated following sciatic nerve injury, but its function has not been investigated (Jang et al., 2010).

Here, we investigated the function of CRMP4 in response to PNS injury. We found that *Crmp4* deletion impaired the regeneration of sensory PNS neurons and delayed Wallerian degeneration of the distal processes *in vitro* and *in vivo*. Degeneration of the distal processes was facilitated by the calpain-mediated generation of toxic CRMP4 fragments. We conclude that in contrast to its affect in CNS neurons, CRMP4 facilitates PNS axon regeneration by coordinately regulating the regrowth of injured axons and Wallerian degeneration of the disconnected distal process.

## Materials and Methods

### Animals

Animal procedures were performed in accordance with the Canadian Council on Animal Care Guidelines and were approved by the McGill University Animal Care and Use Committee. *Crmp4^+/−^* mice were generated and maintained on a C57BL/6J background as described previously (Khazaei et al., 2014). *Crmp4^−/−^* mice and *Crmp4^+/+^* littermate controls were generated by intercrossing *Crmp4^+/−^* mice. *Caspase-3^−/−^* mice were obtained from The Jackson Laboratory (strain B6.129S1-Casp3tm1Flv/J). Embryonic day 15 (E15) to E16 and postnatal day 4 (P4) to P7 C57BL/6 wild-type mice and Sprague Dawley rats were provided by Charles River Laboratories.

### Antibodies

The following antibodies were used for immunostaining and Western immunoblots: rabbit anti-stathmin-2 (STMN2; catalog #NBP1-49 461, Novus Biologicals; RRID:AB_10011569); mouse anti-tubulin β3 (TUBB3; clone TUJ1; catalog #801202, BioLegend; RRID:AB_10063408); TUBB3 (clone TUJ1; catalog #AB9354, Millipore; RRID:AB_570918); purified rabbit anti-tubulin β3 (clone Poly18020; catalog #802001, BioLegend; RRID:AB_2564645); mouse α-tubulin (catalog #T9026, Sigma-Aldrich; RRID:AB_477593); rabbit CRMP4 a/b (prepared in-house; Alabed et al., 2007); mouse α-fodrin (clone AA6; catalog #BML-FG6090, Enzo Life Sciences; RRID:AB_10554860); mouse anti-His antibody (catalog #34 670, QIAGEN; RRID:AB_2571551); anti-GST rabbit antibody (provided by the laboratory of Peter McPherson, Montreal Neurologic Institute); mouse anti-rat CD68 conjugated to Alexa Fluor 647 (catalog #MCA341A647, Bio-Rad; RRID:AB_566874); anti-neurofilament 200 kDa conjugated to Alexa Fluor 555 (clone NE14; catalog #MAB5256A5, Millipore; RRID:AB_2631099); anti-S-100 protein (clone 15E2E2; catalog #MAB079-1, Millipore; RRID:AB_571112); Alexa Fluor 488-conjugated goat anti-mouse antibody (catalog #A11001, Thermo Fisher Scientific; RRID:AB_2534069); fluorescein-conjugated goat anti-rabbit antibody (catalog #F2765, Thermo Fisher Scientific; RRID:AB_2536525); Alexa Fluor 568-conjugated goat anti-rabbit antibody (catalog #A11011, Thermo Fisher Scientific; RRID:AB_143157); Alexa Fluor 568-conjugated goat anti-mouse antibody (catalog #A11031, Thermo Fisher Scientific; RRID:AB_144696); horseradish peroxidase (HRP)-conjugated anti-mouse IgG antibody (catalog #115–035-003, Jackson ImmunoResearch; RRID:AB_10015289); and HRP-conjugated anti-rabbit IgG antibody (catalog #111–035-003, Jackson ImmunoResearch; RRID:AB_2313567).

### Plasmids and mutagenesis

Cloning of pcDNA3 CRMP4S-WT, and pET TAT v1 TAT-RFP were previously described (Alabed et al., 2007; Khazaei et al., 2015). To generate a pcDNA3 CRMP4S–T524A construct, the T524A mutation was introduced in pcDNA3 CRMP4S-WT using the Quik Change II XL Site-Directed Mutagenesis Kit (Agilent Technologies). To create a DNA construct encoding CRMP4 Amino-terminal fragment (NTF) and Carboxy-terminal fragment (CTF), the nucleotide sequence corresponding to amino acids 1–520 or 521–570 of pcDNA3 CRMP4S-WT, respectively, was amplified by PCR. The resulting sequences were introduced in a pET TAT v1 vector or in a pGEX-4T-1 vector using restriction enzymes.

### Purification of TAT peptides

TAT-RFP peptides were produced from Chinese hamster ovary cells, as described previously (Khazaei et al., 2015). To generate TAT-CRMP4 NTF and CTF peptides, BL21 bacterial cultures expressing pET Tat v1 CRMP4-NTF or CRMP4-CTF were induced overnight with 1 mm Isopropyl β- d-1-thiogalactopyranoside (IPTG). The induced cultures were centrifuged, and the pellet was resuspended in ice-cold buffer A (10 mm Tris-HCl, pH 7.5; 600 mm NaCl; 20 mm imidazole; 1× complete protease inhibitors). The cell lysate was sonicated and cleared by ultracentrifugation. The cleared supernatant was applied to a Ni-NTA column equilibrated with buffer A. The column was washed with buffer A, and the proteins were eluted with buffer B (20 mm Tris-HCl pH 7.5, 1 M NaCl, 250 mm imidazole). The buffer in the eluate was exchanged with buffer C (20 mm NaKPO_4_, pH 6.8; 600 mm NaCl; 5% glycerol) using a PD-10 column (GE Healthcare). The eluate was then applied to a 30S IEX column equilibrated with buffer D (10 mm NaKPO_4_, pH 6.8; 300 mm NaCl; 2.5% glycerol). Finally, the column was washed with buffer D and eluted with buffer E (10 mm NaKPO_4_, pH 6.8; 1.5 M NaCl; 2.5% glycerol). The eluate was concentrated on Amicon Ultra-4 3K or 10K Centricon column (EMD Millipore), aliquoted, and stored at −80°C.

### Purification of GST-CRMP4 NTF proteins

The purification of GST-CRMP4 protein was performed as described previously (Khazaei et al., 2014). Briefly, GST-CRMP4 NTF and GST as a control were expressed in *Escherichia coli* BL21 strain by induction with 0.5 mm IPTG. Bacterial pellets were then resuspended in lysis buffer (50 mm Tris-HCl, pH 7.5; 50 mm NaCl; 5 mm MgCl_2_; 1 mm DTT; protease inhibitors) and lysed by sonication (three times at 50% amplitude for 30 s). The clarified lysate was incubated with Glutathione-agarose beads (GE Healthcare Bio-Sciences) for 2 h at 4°C. Beads were then washed in washing buffer (50 mm Tris-HCl, pH 7.5; 150 mm NaCl; 5 mm MgCl_2_; 1 mm DTT) and were eluted with 20 mm glutathione in elution buffer (50 mm Tris-HCl, pH 8.0; 150 mm NaCl; 5 mm MgCl_2_; 1 mm DTT). The eluate was concentrated on an Amicon Ultra-4 30K Centricon column concentrator (Millipore). Samples were aliquoted and stored at −80°C.

### Culture of dissociated dorsal root ganglion neurons

E15-16 and P4-7 rodent dorsal root ganglia (DRGs) were dissected in ice-cold Leibovitz (L-15) medium (Thermo Fisher Scientific). The DRGs were dissociated in 0.25% trypsin-EDTA (Thermo Fisher Scientific) at 37°C, gently triturated with a P1000 pipette tip, and resuspended in DRG media (Neurobasal, Thermo Fisher Scientific), 1% B27 (catalog #17 504–044, Thermo Fisher Scientific), 1% N2 (catalog #17 502–048, Ther-mo Fisher Scientific), 1% Life Technologies penicillin-streptomycin (catalog #15 140–122, Thermo Fisher Scientific), 2 mm Life Technologies l-glutamine (catalog #25 030–081, Thermo Fisher Scientific), 10 μm 5-fluoro-2′-deoxyuridine (catalog #F0503, Sigma-Aldrich) supplemented with 50 ng/ml nerve growth factor (NGF; catalog #CLMCNET-001, Cedarlane). The dissociated DRG neurons were seeded onto culture plates or in microfluidic devices precoated with 100 μg/ml poly-l-lysine (PLL; catalog #P1399, Sigma-Aldrich) and 5–10 μg/ml laminin (catalog #354232, Corning).

### Preparation of nogo-22 and treatment of DRG cultures

Production of Nogo-22 kDa protein was performed as described previously (Huebner et al., 2011). Dissociated P4 to P7 rat DRG neurons were treated for 5 h with 600 ng GST-Nogo-22 or GST as a control. Lysates were prepared by collecting the treated DRGs in RIPA lysis buffer (50 mm Tris-HCl, pH 7.4; 150 mm NaCl; 5 mm EDTA; 1% Triton X-100; 0.1% sodium deoxycholate; 0.1% SDS; 1 mm Na_3_VO_4_; 5 mm NaF; and 1× complete protease inhibitors). The protein concentration in the lysates was normalized prior to analysis by Western blot.

### *In vitro* regeneration of DRG axons in microfluidic devices

Dissociated E15-16 mouse DRG neurons were seeded at a density of 10,000–15,000 neurons in microfluidic neuro devices (ANANDA Devices) adhered to 3.5 cm imaging dishes (MatTek) precoated with 100 μg/ml PLL and 5 μg/ml laminin. At 4 d *in vitro* (DIV), the axons projecting to the bottom compartment were stained with 4 μl of 10 μm Invitrogen Mitotracker Green FM (catalog #M7514, Thermo Fisher Scientific) for 30 min. They were then axotomized by increasing the flow of medium in the bottom compartment of the microfluidic device using simultaneous vacuum aspiration and medium replacement to shear the axons. At 24 h following the axotomy, the neurons were fixed and stained with anti-TUBB3 antibody (1:1000) and Alexa Fluor 568-conjugated secondary antibody (1:1000). Fluorescent images of the samples were acquired using an Axiovert1 microscope (Zeiss) with a 20× objective (Plan-APOCHROMAT Pln Apo 20×/0.8; Zeiss). Regrowth of the axons after axotomy was measured by manually tracing the axons with the NeuronJ plugin for ImageJ (Meijering et al., 2004). The extent of regeneration was measured by dividing the total neuronal regrowth in the bottom compartment of the microfluidic device by the number of channels with growing axons.

### Treatment of DRG neurons grown in microfluidic devices with TAT- or GST-CRMP4 peptides

Dissociated E15-16 mouse DRG neurons were seeded at a density of 17,000–25,000 neurons in microfluidic neuro devices (ANANDA Devices) adhered to a 3.5 cm imaging dish (MatTek) precoated with 100 μg/ml PLL and 5 μg/ml laminin. At 3 DIV, the axons projecting to the bottom compartment of the microfluidic devices were treated for 8 h with 2.5 μm TAT-CRMP4-His-V5 peptides, GST-CRMP4 NTF peptides, or with TAT-RFP or GST as controls. The neurons were then fixed, and stained with anti-TUBB3 rabbit antibody (1:1000), anti-His mouse antibody (1:500), anti-GST rabbit antibody (1:500), and Alexa Fluor-conjugated secondary antibodies (1:1000). Fluorescent images of the samples were acquired using an Axiovert 1 Microscope (Zeiss) with a 20× objective (Plan-Apochromat Pln Apo 20×/0.8; Zeiss). The extent of degeneration was measured in at least three representative regions of interest (400 × 400 μm) per explant by calculating the index of degeneration, which corresponds to the area covered by the axonal fragments with a circularity >0.9 divided by the total area covered by the axons (Kilinc et al., 2011).

### Axotomy-induced degeneration of E12.5 DRG explants

Axotomy-induced degeneration of E12.5 mouse DRG explants was performed as previously described (Unsain et al., 2014). Briefly, the DRG explants were seeded onto cell-filter inserts coated with 1 mg/ml PLL, 10 μg/ml laminin, and 0.1 mg/ml collagen, filled with DRG media with the bottom compartment supplemented with 15 ng/ml NGF. After ∼60 h of growth, the explants on the upper side of the cell-filter inserts were detached with a cell scraper, and the axotomized axons were left to degenerate for 3 h, after which they were collected in Laemmli sample buffer for subsequent analysis by Western blot. In some experiments, the DRG explants were treated with 5–15 μm ALLN (catalog #208719, EMD Millipore) prior to the axotomy. Alternatively, the DRG explants were grown on coated cell culture plates containing DRG media supplemented with 10 ng/ml NGF for ∼60 h. The axons of the DRG explants were sectioned with a scalpel blade, and the axotomized axons were allowed to degenerate for 5–5.5 h. The DRG explants were then fixed and stained with anti-TUBB3 antibody (1:1000) and Alexa Fluor 568-conjugated secondary antibody (1:1000). The axotomized DRG explants were imaged with an Axiovert 200 M Microscope (Zeiss) using a 10× objective (CP-ACHROMAT 10×/0.25 Ph1; Zeiss). The extent of degeneration was measured in at least three representative regions of interest per explant (400 × 400 μm) by calculating the index of degeneration, which corresponds to the area covered by the axonal fragments with a >0.9 divided by the total area covered by the axons (Kilinc et al., 2011).

### NGF withdrawal from E12.5 DRG explants

This experiment was conducted as previously described using E12.5 mouse explants (Unsain et al., 2014). Briefly, the E12.5 DRG explants were seeded into 6-well plates precoated with 1 mg/ml PLL, 10 μg/ml laminin and 0.1 mg/ml collagen in DRG media supplemented with 10 ng/ml NGF. After ∼2 d of growth, the media was exchanged to NGF-free DRG media supplemented with 10 μg/ml anti-NGF antibodies. NGF withdrawal-induced degeneration was allowed to proceed for 24 h, after which the NGF-deprived axons were fixed and stained with anti-TUBB3 antibody (1:10 000) and Alexa Fluor 568-conjugated antibody (1:5000). The axons were imaged at a 5× magnification using a Zeiss Axioscope 2 Microscope. The quantification of the area covered by the axons was done with Axoquant 2.0 in R Studio as previously described (Johnstone et al., 2018).

### Immunostaining of dissociated DRG neurons and DRG explants

The dissociated DRG neurons or DRG explants were fixed for 30 min with 4% paraformaldehyde (PFA) diluted in PBS then thoroughly washed with PBS. The neurons were then permeabilized for 5 min with 0.2% Triton X-100/PBS prior to blocking in 5% bovine serum albumin (BSA) diluted in PBS for 1 h at room temperature. The neurons were then stained with primary antibody diluted in 5% BSA/PBS overnight at 4°C. The next day, the samples were washed in PBS, incubated in secondary antibody diluted in 5% BSA/PBS for 2 h at room temperature, and washed again in PBS.

### Sciatic nerve injury

Male and female mice 3–4 months old were used for the sciatic nerve injuries. Following anesthesia with isoflurane, the sciatic nerve was exposed with a mid-thigh incision and crushed with a smooth-jaw hemostat (0.6 mm tip; Fine Science Tools) fully closed for 30 s at ∼1 cm distal to the sciatic notch. The injury site was labeled by attaching a 9–0 nonabsorbable silk suture (Ethicon) to the epineurium. Alternatively, the sciatic nerve was transected with straight semifine scissors (Fine Science Tools), and the nerve ends were pulled apart to prevent regeneration. Analgesia was managed by injecting buprenorphine (0.10 mg/kg) subcutaneously and providing carprofen-containing MediGel CPF (ClearH_2_O) *ad libitum*. Animals were also given enrofloxacin (5 mg/kg, s.c.) preoperatively and postoperatively to prevent infection.

### Optic nerve injury

Male and female mice 2–3 months old were used for optic nerve transection. Following anesthesia with isoflurane, the optic nerve was exposed with an incision above the ocular orbit and the extraocular muscles were resected. The optic nerve was transected with semifine scissors (Fine Science Tools) at 0.5–1.0 mm from the optic nerve head. Care was taken to avoid damage to the ophthalmic artery, and the vascular integrity of the retina was assessed by fundus examination. Analgesia was provided by subcutaneous injections of buprenorphine (0.05 mg/kg).

### *In vivo* analysis of neuronal regeneration in whole-mount stained sciatic nerves

At 3 d postinjury (DPI), mice were killed with isoflurane and CO_2_ inhalation followed by cervical dislocation. The injured and contralateral intact sciatic nerves were harvested and fixed in 4% PFA/PBS for 5 h at 4°C. Following fixation, the tissues were washed in PBS, then dehydrated in sequential washes of 50%, 80%, and 100% methanol (Sigma-Aldrich) for 1 h each at room temperature, before quenching the endogenous peroxidase activity overnight at 4°C with ice-cold H_2_O_2_ diluted in 20% DMSO/methanol (1 vol of 30% H_2_O_2_, 1 vol of DMSO, 4 vol of methanol). The following day, the tissues were rehydrated in a reversed gradient of methanol followed by two washes of 1 h in PBS. Blocking was performed overnight at 4°C in blocking buffer [10% normal goat serum (NGS), 10% DMSO (Sigma-Aldrich), and 0.2% Triton X-100 (Sigma-Aldrich), PBS]. The sciatic nerves were then stained sequentially with anti-STMN2 [also referred to as superior cervical ganglion 10 (SCG10) 1:200] primary antibody and Alexa Fluor 568-conjugated goat anti-rabbit secondary antibody (1:200) diluted in antibody dilution buffer (3% NGS, 5% DMSO, 0.2% Triton X-100, 10 10 μg/ml heparin, PBS) for 7 d at 37°C to label the regenerating sensory axon front as the anti-STMN2/SCG10 antibody preferentially detects regenerating sensory and not motor axons (Shin et al., 2014). Following each incubation, the nerves were thoroughly washed in 0.2% Triton X-100/PBS for 7 h at room temperature, changing the wash buffer every hour. A final overnight wash was done in PBS before tissue clarification. Prior to imaging, the stained nerves were cleared following an adapted 3DISCO protocol (Ertürk et al., 2012). Briefly, the nerves were dehydrated in a stepwise gradient of 50%, 80%, and 100% tetrahydrofuran (Sigma-Aldrich) diluted in distilled water. The nerves were then cleared by immersion in dibenzyl ether (Sigma-Aldrich). The whole nerves were imaged with an SP8 confocal laser-scanning microscope (Leica) using a 10× objective (HC PL APO 1×/0.40 CS). The distance of regeneration was measured as described previously (Leon et al., 2000). Briefly, the number of SCG10^+^ neurons located at 500 μm increments from the injury site were counted in alternating optical sections. The resulting number was divided by the diameter of the nerve (in millimeters) to calculate the number of axons per millimeter for each section counted. The number of axons/millimeter was then averaged over all the sections. Finally, the total number of axons $\sum a_{d}$ extending to a distance *d* was estimated by summing all sections:

$$\sum a_{d}=\pi r^{2}\;[average\;axons/mm]/t$$

where *r* is the radius of the nerve and *t* is the optical thickness of the sections.

### *In vivo* analysis of axonal degeneration in sciatic nerves

The mice were killed by intracardial perfusion with ice-cold 4% PFA. The nerve stump distal to the injury site and the corresponding contralateral nerve were harvested and postfixed in 2.5% gluteraldehyde in 0.1 m PBS. Postprocessing of the nerve samples was conducted by the Facility for Electron Microscopy Research (FEMR) of McGill University. Briefly, the fixed sciatic nerves were stained with 1% osmium tetroxide, dehydrated in sequential washes with a gradient of ethanol and embedded in epoxy embedding medium (Electron Microscopy Sciences). Semithin transverse nerve sections with a thickness of 0.5 μm were prepared using an ultramicrotome (Reichert) and mounted with a coverslip using Permount mounting medium (catalog #SP15-100, Fisher Scientific). Imaging was performed with an Axiovert 200 M Epifluorescence Microscope (Zeiss) using a 63× objective (Plan-Neofluar 1.25 Oil; Zeiss). To evaluate the extent of degeneration, the number of intact axons per square micrometer was counted.

### Tissue homogenization

At selected time points following nerve transection, the mice were killed with isoflurane and CO_2_ inhalation followed by cervical dislocation. Sciatic nerve, optic nerve, L4–L6 DRGs and retina samples were collected; washed briefly in ice-cold PBS; and manually homogenized in RIPA lysis buffer (50 mm Tris-HCl, pH 7.4; 150 mm NaCl; 5 mm EDTA; 1% Triton X-100; 0.1% sodium deoxycholate; 0.1% SDS; 1 mm Na_3_VO_4_; 5 mm NaF; and 1× complete protease inhibitors). The protein concentration in the resulting sonicated and cleared lysates was measured and normalized between samples prior to analysis by Western immunoblot.

### Overexpression of CRMP4 in HEK 293T cells and *in vitro* digestion with recombinant calpain

HEK293T cells maintained in DMEM medium supplemented with 10% fetal bovine serum were transfected using Invitrogen Lipofectamine 2000 (Thermo Fisher Scientific) following manufacturer instructions. The cells were washed briefly in ice-cold PBS and lysed with RIPA lysis buffer (50 mm Tris-HCl, pH 7.4; 150 mm NaCl; 5 mm EDTA; 1% Triton X-100; 0.1% sodium deoxycholate; 0.1% SDS; 1 mm Na_3_VO_4_; 5 mm NaF; and 1× complete protease inhibitors). The protein concentration in the sonicated and cleared lysates was normalized. The lysates were digested *in vitro* with 1 unit of recombinant calpain-1 (catalog #208713, EMD Millipore) and 2 mm MgCl_2_ for 30 min at 37°C. The digested proteins were then analyzed by Western blot. In some experiments, the calpain inhibitor 5–15 μm ALLN (catalog #208719, EMD Millipore) was added to the reaction.

### Western blots

The protein content of tissue and cell lysates was analyzed by SDS-PAGE gel separation followed by Western immunoblot. PVDF membranes were blocked with 5% milk diluted in Tris-buffered saline supplemented with 0.05% Triton X-100 for 1 h at room temperature, and probed with primary antibody overnight at 4°C and with secondary antibodies for 1 h at room temperature. The antibodies used were as follows: rabbit CRMP4 a/b (1:7500), mouse α-fodrin (1:500), mouse anti-tubulin β3 (1:5000), mouse α-tubulin (1:5000), and HRP-conjugated anti-mouse and anti-rabbit IgG antibodies (1:10,000). The signal was revealed with Western Lightning Plus ECL (PerkinElmer). Quantification of the intensity of the bands was performed in Photoshop. Changes in CRMP4 expression in the cell body compartment was normalized to a tubulin loading control.

### Statistical analysis

Statistical analyses were performed with GraphPad Prism 8 software. Two-tailed Student’s *t* test were used when directly comparing two conditions, while one-way ANOVA followed by Tukey’s multiple-comparison tests were used when more than three conditions were compared. For experiments with more than one variable parameter such as the *in vivo* regeneration and degeneration experiments, a two-way ANOVA followed by Bonferroni’s multiple-comparison tests were used (Table 1). Statistical details, *p* values, and experimental details are indicated in the corresponding figure legends.

**Table 1:** Statistical tests

Figure	Data structure	Type of test	Sample size	Statistical data
Figure 1*B*	Two factors (genotype and distance of regeneration)	Two-way ANOVA	CRMP4^+/+^ mice: *n* = 6; CRMP4^−/−^ mice: *n* = 5	Distance: *F*_(13,126)_ = 18.95; *p* < 0.0001; genotype: *F*_(1,126)_ = 12.49; *p* = 0.0006; interaction genotype/distance: *F*_(13,126)_ = 0.5537; p = 0.8860; Sidak’s multiple comparison test: CRMP4^+/+^ vs CRMP4^−/−^ at all distances from the injury site: n.s.
Figure 3*C*	Normal distribution	Unpaired Student t-test (two-tailed)	Wild-type DRG neurons: *n* = 8; CRMP4^−/−^ DRG neurons: *n* = 7	*t* = 4.105; df = 13; *p* = 0.0012
Figure 4*B*	Two factors (genotype and duration of degeneration)	Mixed effects analysis	For CRMP4^+/+^ and CRMP4^−/−^ mice: Intact sciatic nerves: *n* = 7; Transected sciatic nerves at 36 HPI: *n* = 4; Transected sciatic nerves at 3DPI and 7DPI: *n* = 3	Time postinjury: *F*_(3,14)_ = 110.5; genotype: *F*_(1,12)_ = 8.569; *p* = 0.0127; interaction genotype/time postinjury: *F*_(3,14)_ = 1.768; *p* = 0.1994; Sidak’s multiple comparison test: intact CRMP4^+/+^ vs CRMP4^−/−^ nerves: n.s.; transected CRMP4^+/+^ vs CRMP4^−/−^ nerves at 36 HPI: n.s.; transected CRMP4^+/+^ vs CRMP4^−/−^ nerves at 3 DPI: *p* = 0.0016; Transected CRMP4^+/+^ vs CRMP4^−/−^ nerves at 7 dpi: n.s.
Figure 4*D*	Normal distribution	Unpaired Student t-test (two-tailed)	CRMP4^+/+^ explants: *n* = 10; CRMP4^−/−^ explants: *n* = 9	*t* = 2.311; df = 17; *p* = 0.0336
Figure 6*E*	Normal distribution	One-way ANOVA	Wild-type DRG neurons: *n* = 4	*F* = 17.15; *p* = 0.0001; *R*^2^ = 0.8109; Tukey’s multiple comparison test: nontreated vs TAT-RFP: n.s.; nontreated vs TAT-CRMP4 NTF: *p* = 0.0006; nontreated vs TAT-CRMP4 CTF: *p* = 0.0105; TAT-RFP vs TAT-CRMP4 NTF: *p* = 0.0003; TAT-RFP vs TAT-CRMP4 CTF: *p* = 0.0053; TAT-CRMP4 NTF vs TAT-CRMP4 CTF: n.s.
Figure 6*F*	Normal distribution	One-way ANOVA	Wild-type DRG neurons: *n* = 3	*F* = 15.06; *p* = 0.0012; *R*^2^ = 0.8496; Tukey’s multiple comparison test: TAT-RFP vs TAT-CRMP4 NTF: *p* = 0.0017; TAT-RFP vs GST: n.s.; TAT-RFP vs GST-CRMP4 NTF: n.s.; TAT-CRMP4 NTF vs GST: *p* = 0.0037; TAT-CRMP4 NTF vs GST-CRMP4 NTF: *p* = 0.0032; GST vs GST-CRMP4 NTF: n.s.
Figure 4*G*	Normal distribution	One-way ANOVA	CRMP4^+/+^ DRG explants: *n* = ; CRMP4^−/−^ DRG explants: *n* = 4	*F* = 24.79; *p* < 0.0001; *R*^2^ = 0.8230; Tukey’s multiple-comparison test: CRMP4^+/+^+NGF vs CRMP4^+/+^-NGF: *p* = 0.0024; CRMP4^+/+^+NGF vs CRMP4^−/−^+NGF: n.s.; CRMP4^+/+^+NGF vs CRMP4^−/−^-NGF: *p* = 0.0025; CRMP4^+/+^-NGF vs CRMP4^−/−^+NGF: *p* = 0.0052; CRMP4^+/+^-NGF vs CRMP4^−/−^-NGF: n.s.; CRMP4^−/−^+NGF vs CRMP4^−/−^-NGF: *p* = 0.0048

## Results

### Crmp4 deletion impairs regeneration of sensory neurons following sciatic nerve injury

**Figure 1.**
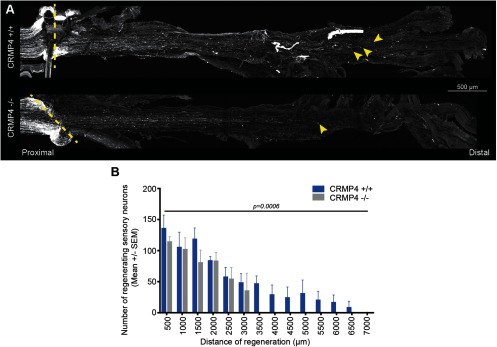
*Crmp4* deletion impairs the regeneration of sensory neurons following sciatic nerve injury. ***A***, Maximal intensity projections of straightened *Crmp4^+/+^ or Crmp4 ^−/−^* sciatic nerves at 3 DPI following crush injury stained with SCG10, a marker of the regenerating sensory neurons. The injury site is labeled by a dotted line, and the extremity of the regenerating fibers is indicated by arrowheads. Scale bar, 500 μm. ***B***, Quantification of the distance of regeneration of the SCG10^+^ sensory neurons at 3 DPI in *Crmp4*^+/+^ and *Crmp4^−/−^* mice. Data are represented as the mean number of neurons per square millimeter ± SEM. The difference between the genotypes is statistically significant (*n* = 5–6; two-way ANOVA, *p* = 0.0006).

Paradoxically, CRMP4 facilitates growth during development, but impedes regeneration of injured CNS neurons (Quinn et al., 2003; Alabed et al., 2007; Nagai et al., 2012, 2015, 2016; Niisato et al., 2012; Khazaei et al., 2014; Tan et al., 2015). Thus, we sought to investigate the functions of CRMP4 in regeneration-competent PNS neurons. Sciatic nerve crush injuries were performed on adult *Crmp4^−/−^* mice and littermate *Crmp4^+/+^* controls. Regenerating fibers were stained for SCG10, a protein that is preferentially upregulated in regenerating sensory neurons, and their growth was evaluated at 3 DPI (Mason et al., 2002; Shin et al., 2014). In both *Crmp4^+/+^* and *Crmp4^−/−^* sciatic nerves, SCG10^+^ neurons grew spontaneously across the injury site following sciatic nerve crush injury (Fig. 1*A*). However, quantification of the number of regenerating fibers at progressive distances from the lesion site revealed that sensory neurons in *Crmp4^+/+^* mice extend further than those in *Crmp4^−/−^* mice (Fig. 1*B*), revealing a proregenerative function for CRMP4 in the PNS, which contrasts to its inhibitory role in the CNS (Alabed et al., 2007; Nagai et al., 2012, 2015, 2016).

### CRMP4 expression is differentially regulated in the CNS and PNS following injury

**Figure 2.**
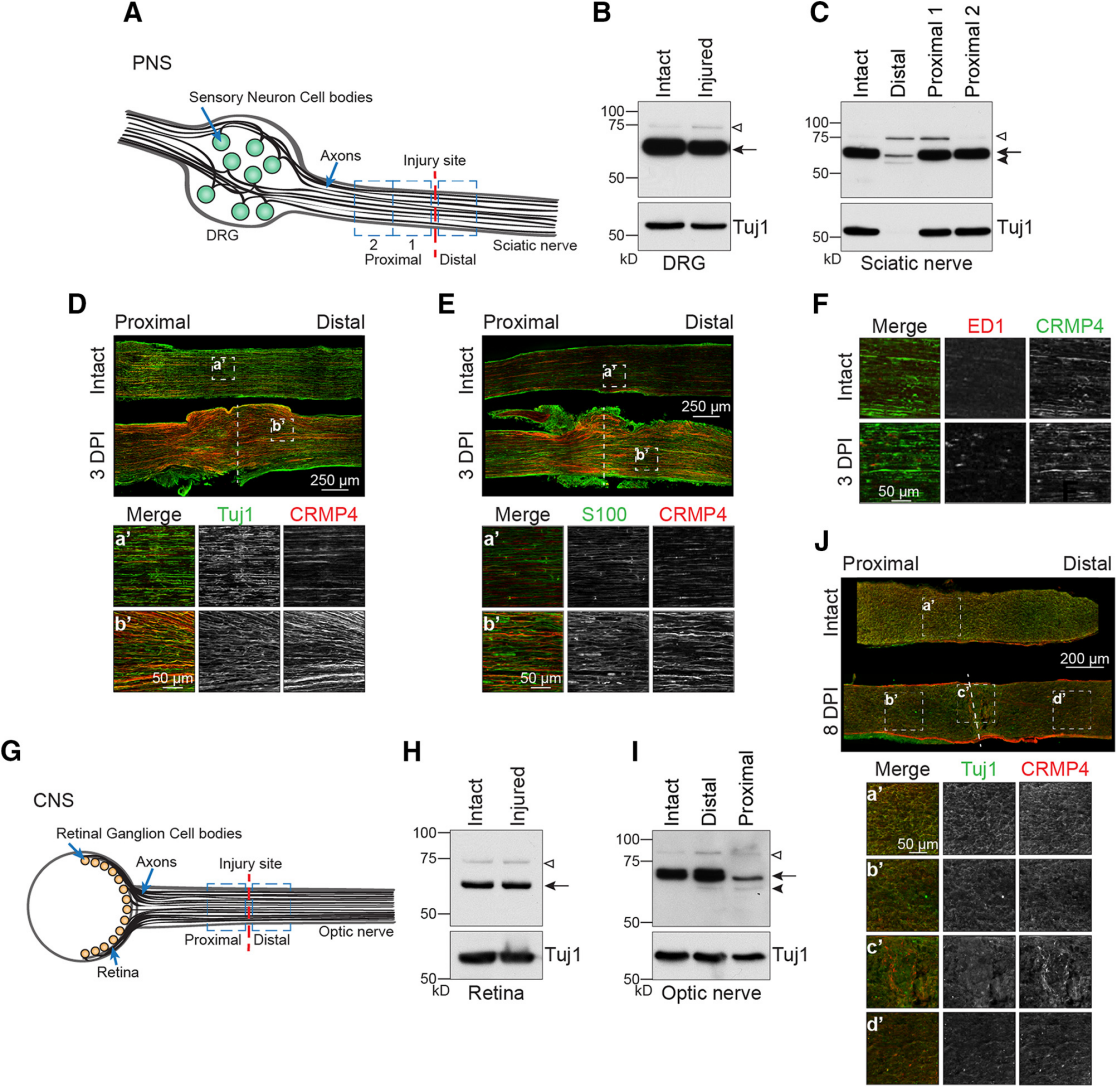
The expression of CRMP4 is differentially regulated following CNS and PNS injury. ***A***, Illustration of the sciatic nerve injury model used to examine the response to PNS injury. ***B***, ***C***, Immunoblot analysis of the CRMP4 expression pattern in DRG (***B***) and sciatic nerve (***C***) lysates from wild-type mice at 3 d following sciatic nerve transection. The nerve samples collected span 0–3 mm distal, 0–3 mm proximal (proximal 1), or 3–6 mm proximal (proximal 2) to the injury site. The data are representative of results obtained from seven mice. Open arrowhead, CRMP4L (75 kDa); arrow, CRMP4S (65 kDa); solid arrowhead, tCRMP4 (55 kDa). ***D*–*F***, Representative images of longitudinal sections of intact and injured sciatic nerves to evaluate the colocalization of CRMP4 with Tuj1 (***D***), S100 (***E***), and ED1 (***F***), which are, respectively, markers of neurons, Schwann cells, and macrophages. ***G***, Diagram of the optic nerve injury model used to examine the response to CNS axotomy. ***H***, ***I***, Immunoblot analysis of CRMP4 expression in retina (***H***) and optic nerve (***I***) lysates at 3 d following optic nerve transection. The nerve portions analyzed span from either 0–2 mm distal or 0–2 mm proximal to the injury site in the optic nerves. The data are representative of four independent replicates. Open arrowhead, CRMP4L (75 kDa); arrow, CRMP4S (65 kDa); solid arrowhead, tCRMP4 (55 kDa). ***J***, Representative images of longitudinal sections of intact and injured optic nerves to evaluate the colocalization of CRMP4 a/b with the neuronal marker Tuj1.

Next, we sought to determine whether the distinct regenerative phenotypes observed in the PNS and CNS could stem from differences in the expression profile of CRMP4. CRMP4 expression was assessed by Western blot analysis of lysates prepared from PNS and CNS cell bodies and axons. For analysis of the PNS, lumbar DRGs containing the cell bodies of sensory neurons and sciatic nerves containing their axons were collected at 3 d following sciatic nerve transection (Fig. 2*A*). CRMP4 was robustly expressed in intact and injured DRGs with little change in expression following injury (Fig. 2*B*; CRMP4S expression, 88 ± 12% control). In the sciatic nerve, expression of the CRMP4S was markedly reduced in the nerve distal to the lesion site where axons undergo Wallerian degeneration (56 ± 1% of control levels), mirroring the regulation of tubulin (33 ± 8% of control), and bands representing the CRMP4L and a truncated 55 kDa CRMP4 cleavage fragment (tCRMP4) became apparent (Fig. 2*C*). In the proximal segment of sciatic nerve where the growth cones of regenerating neurons reform, CRMP4S expression remained constant (94 ± 5% and 99 ± 3% in proximal 1 and proximal 2 segments, respectively) and CRMP4L was modestly upregulated (Fig. 2*C*). To determine the cell types expressing CRMP4, crushed sciatic nerves were stained with a pan-CRMP4 antibody. CRMP4 colocalized with the neuronal marker Tuj1 (Fig. 2*D*), but not with the Schwann cell marker S100 (Fig. 2*E*) or the macrophage marker ED1 (Fig. 2*F*). This reveals that CRMP4 is preferentially expressed in neurons, suggesting a neuron cell-autonomous role following PNS injury.

We then analyzed the expression of CRMP4 in the damaged CNS, focusing on the retinal ganglion cells and the retina at 3 d after optic nerve transection (Fig. 2*G*). In the CNS, CRMP4 expression was unaltered in the retina 3 d following optic nerve transection (Fig. 2*H*; CRMP4S expression 101 ± 6% of control levels), similar to the CRMP4 expression pattern observed in DRG cell bodies. In the optic nerve, CRMP4 regulation was notably different from the sciatic nerve (Fig. 2*I*). In the optic nerve segments, truncated CRMP4 was apparent in the distal segment following optic nerve injury, but CRMP4S and CRMP4L expression was largely retained (CRMP4S, 100 ± 1% of control). In the proximal segment of the damaged optic nerve, CRMP4S was strongly downregulated (31 ± 9% of control levels) with the appearance of tCRMP4 (Fig. 2*I*). Intriguingly, this reveals a distinct regulation of CRMP4 following injury to PNS versus CNS neurons. In the regenerating PNS, CRMP4 expression is retained in the proximal segment of sciatic nerve correlating with the regeneration of severed axons, whereas CRMP4 is downregulated in the proximal segment of optic nerve where axons fail to regrow. Further, in the distal sciatic nerve, CRMP4 is cleaved and downregulated, coinciding with the onset of Wallerian degeneration, while CRMP4 expression is largely retained at the same time point in the distal optic nerve. CRMP4 is also expressed in different cell types in the crushed optic nerve at 8 DPI, as it is expressed in the scar-forming non-neuronal cells surrounding the injury site (Fig. 2*J*). The distinct regulation of CRMP4 in injured PNS and CNS neurons led us to speculate that full-length CRMP4 in the proximal segment of injured PNS neurons may support regrowth, whereas CRMP4 cleavage may contribute to Wallerian degeneration in the distal segment of sciatic nerve.

### CRMP4 enhances regrowth of injured sensory neurons *in vitro*

**Figure 3.**
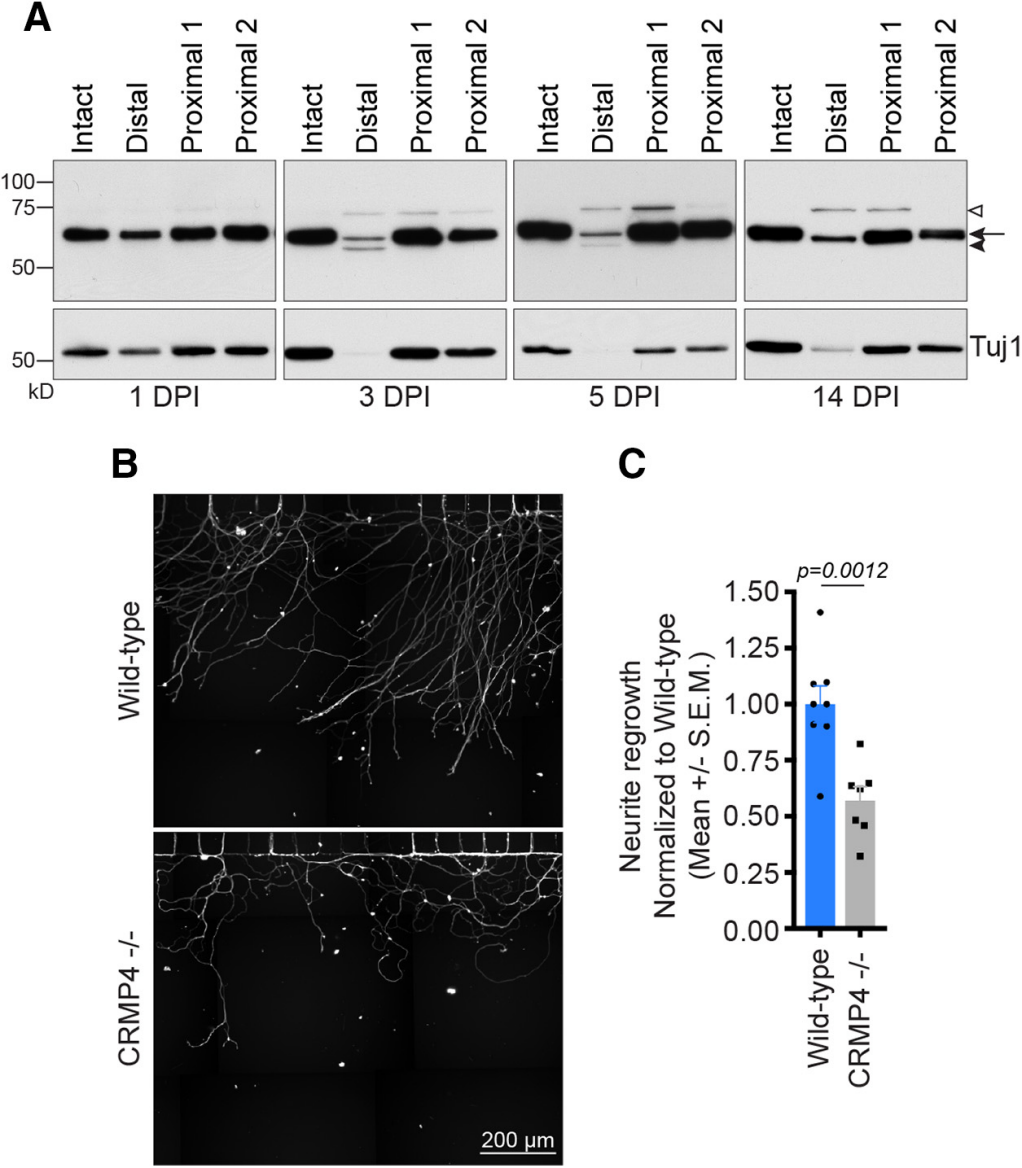
CRMP4 promotes growth of injured sensory neurons. ***A***, Immunoblot analysis of CRMP4 expression in sciatic nerve lysates of wild-type mice at different times following sciatic nerve transection. Samples collected after sciatic nerve transection span 0–3 mm distal, 0–3 mm proximal (proximal 1), or 3–6 mm proximal (proximal 2) to the injury site. Open arrowhead, CRMP4L (75 kDa); arrow, CRMP4S (65 kDa); solid arrowhead, tCRMP4 (55 kDa). The data are representative of results obtained from seven mice. ***B***, ***C***, Representative pictures (***B***) and quantification (***C***) of the regrowth of dissociated E15-16 wild-type or *Crmp4^−/−^* DRG neurons plated in microfluidic devices at 24 h after axotomy indicated an impaired regrowth after axotomy in the *Crmp4^−/−^* DRG neurons. Data are represented as the mean neurite outgrowth per channel ± SEM (*n* = 7–8, two-tailed Student *t* test). Scale bar, 200 μm.

To test whether CRMP4 expression in the proximal segment of injured sensory neurons promotes axon extension after injury, we conducted a more extensive time course analysis of CRMP4 expression following sciatic nerve injury. Following sciatic nerve transection, CRMP4 expression is retained in the proximal nerve segment for up to 14 DPI (Fig. 3*A*). To test the cell-autonomous contribution of CRMP4 to the regrowth of damaged axons, we measure the extent of regeneration of axotomized DRG neurons *in vitro*. DRGs were isolated from E15-16 wild-type or *Crmp4^−/−^* mouse embryos, and the dissociated neurons were plated in microfluidic devices for 4 d until the axons projected into the axonal compartment (Jones et al., 2006; Frey et al., 2015; Dubový et al., 2018). Axotomy was then performed by increasing the flow of medium in the axonal compartment to shear the axons, and the damaged neurons were left to regenerate for 24 h. Following axotomy, *Crmp4^−/−^* DRG neurons regrew significantly worse than their wild-type counterparts, exhibiting 43% less regrowth (Fig. 3*B*,*C*). The regenerating CRMP4*^−/−^* neurites also adopted a curled phenotype, often looping back toward the channels, which differs from the straight outgrowth profile of wild-type neurons (Fig. 3*B*). This finding demonstrates that CRMP4 enhances the regrowth of the proximal tip of axotomized sensory neurons in a neuron cell-autonomous manner.

### Crmp4 deletion delays Wallerian degeneration in the PNS

**Figure 4.**
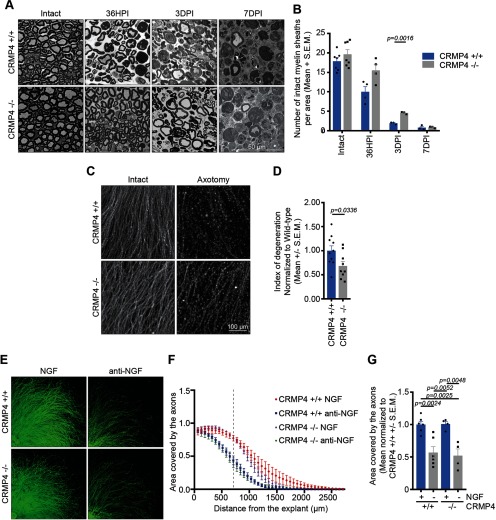
*Crmp4* deletion delays Wallerian degeneration *in vivo* and *in vitro*. ***A***, Semithin cross-sections of the degenerating sciatic nerve at 36 HPI, 3 DPI, or 7 DPI following sciatic nerve transection, or of the intact contralateral nerve, in *Crmp4*^+/+^ or *Crmp4^−/−^* mice. Scale bar, 50 μm. ***B***, Quantification of the mean number of intact myelin sheaths per square micrometer ± SEM in the *Crmp4^−/−^* sciatic nerves compared with the *Crmp4*^+/+^ controls displayed in ***A*** (*n* = 3–7; two-way ANOVA with Bonferroni’s multiple-comparison test). ***C***, ***D***, Representative pictures of the axons extending from DRG explants at a distance of 500 μm from the center of the explant stained with Tuj1 (***C***) and quantification (***D***) of the extent of degeneration in E12.5 *Crmp4*^+/+^ or *Crmp4^−/−^* DRG explants at 5 h after axotomy. Data in the graph are represented as the mean index of degeneration ± SEM (*n* = 9–10; two-tailed Student *t* test). Scale bar, 100 μm. ***E***, Representative images of intact E12.5 DRG explants or at 24 h following NGF withdrawal (anti-NGF). ***F***, Curves of the area covered by axons of *Crmp4^+/+^* and *Crmp4^−/−^* DRG explants in the presence (NGF) or absence (anti-NGF) of NGF. The curves represent the mean ± SEM. ***C***, Mean ± SEM of the area covered by the axons at a distance of 700 μm from the center of the explants (as indicated by a dotted line in ***G***) in the presence or absence of NGF. Data are normalized to *Crmp4^+/+^* grown in the presence of NGF (*n* = 3; one-way ANOVA, Tukey’s multiple-comparison test).

We next investigated whether CRMP4 might be involved in the regulation of Wallerian degeneration in the sciatic nerve. Previous studies have demonstrated that axon regeneration is impaired in mice with delayed Wallerian degeneration, suggesting that clearance of axon distal segments facilitate axon regeneration (Bisby and Chen, 1990; Brown et al., 1992, 1994). We first assessed Wallerian degeneration in the transected sciatic nerve by quantifying the number of intact myelin sheaths in semithin cross sections of the nerve segment located distal to the injury site, an indirect measure of axon degeneration as Wallerian degeneration is followed by the degradation of myelin sheaths. The data revealed a higher number of intact myelinated sheaths in the sciatic nerve collected from *Crmp4^−/−^* mice compared with the *Crmp4^+/+^* controls at 36 h postinjury (HPI), which became statistically significant at 3 DPI (Fig. 4*A*,*B*). By 7 DPI, the degradation of myelin sheaths was extensive in both *Crmp4^+/+^* and *Crmp4^−/−^* sciatic nerves. These results indicate that *Crmp4* deletion delays Wallerian degeneration following sciatic nerve injury.

To better understand the function of CRMP4 in Wallerian degeneration, we examined axon degeneration in an *in vitro* axotomy model (Gerdts et al., 2013; Gamage et al., 2017). E12.5 *Crmp4^+/+^* and *Crmp4^−/−^* DRG explants were grown for 60 h prior to the neurites being sectioned with a blade and allowed to degenerate for 5 h. The index of degeneration of these neurites was then calculated by dividing the area covered by particles with a circularity >0.9, which corresponds to the axon fragments, by the total area covered by the neurites (Kilinc et al., 2011). The quantification revealed that *Crmp4^−/−^* neurons have a lower index of degeneration compared with the *Crmp4^+/+^* neurons, illustrating a cell-autonomous role for CRMP4 in Wallerian degeneration (Fig. 4*C*,*D*). Interestingly, the prodegenerative function of CRMP4 following axotomy is not conserved in another model of degeneration: trophic factor deprivation. When E12.5 *Crmp4^−/−^* and *Crmp4^+/+^* DRG explants were deprived of NGF and treated with anti-NGF antibody, they exhibited typical signs of degeneration, such as the appearance of axon swellings and fragments (Fig. 4*E*). The area covered by the neurites following degeneration was not significantly different between the NGF-deprived *Crmp4^−/−^* and *Crmp4^+/+^* DRG explants (Fig. 4*F*,*G*), indicating that the effect of CRMP4 on Wallerian degeneration is somewhat selective.

### CRMP4 is cleaved by calpain in axotomized sensory neurons

**Figure 5.**
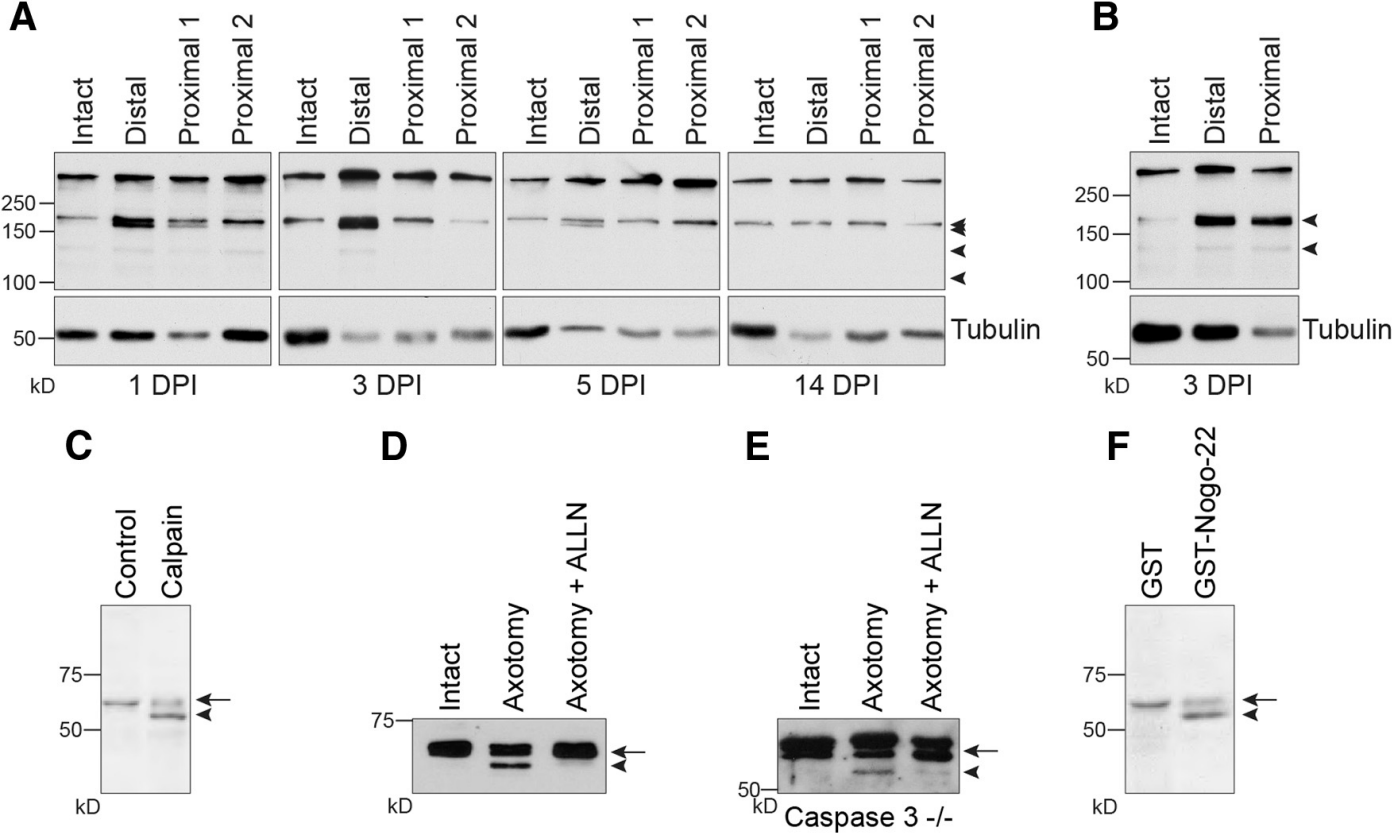
Calpain-dependent cleavage of CRMP4 occurs in degenerating axons following injury. ***A***, ***B***, Immunoblot analysis of the calpain substrate fodrin in sciatic nerve (***A***) and optic nerve (***B***) lysates collected at the indicated DPI. The arrowheads indicate the presence of fodrin breakdown products, which are present when calpain is active. ***C***, Western immunoblot analysis of CRMP4 expression in lysates prepared from P4 to P7 rat DRG neurons and treated with calpain *in vitro*. ***D***, ***E***, Western immunoblot analysis of CRMP4 expression in axonal lysates prepared from wild-type (***D***) or *Caspase-3^−/−^* (***E***) E12.5 DRG explants grown on cell-filter inserts collected at 3 h after axotomy. DRG explants were treated with ALLN to inhibit calpain. ***F***, Western immunoblot analysis of CRMP4 expression in lysates prepared from P4 to P7 rat DRG neurons that were treated for 5 h with either GST or GST-Nogo-22. Arrow, CRMP4S (65 kDa); solid arrowhead, tCRMP4 (55 kDa).

We found that the expression of CRMP4 was downregulated in degenerating axons located distal to a sciatic nerve injury, coincident with the appearance of a truncated CRMP4 species. We thus sought to investigate the mechanism underlying the cleavage of CRMP4 and its contribution to Wallerian degeneration. CRMP family members, including CRMP4, have been described as calpain substrates in response to ischemic and excitotoxic stimuli (Kowara et al., 2005, 2006; Jiang et al., 2007; Zhang et al., 2007; Liu et al., 2009). To determine whether calpain is active in the injured sciatic nerve, we investigated the cleavage of fodrin, a calpain substrate that is alternatively referred to as αII-spectrin and that is often used as a marker of calpain activity (Wang, 2000). Following injury, 145 and 150 kDa spectrin breakdown products characteristic of calpain-mediated cleavage were elevated in the distal sciatic nerve segment, but largely unregulated in the proximal segment (Fig. 5*A*; Wang, 2000). The fragments were detected at 1, 3, and 5 DPI and were resolved by 14 DPI, which is consistent with the time course of CRMP4 cleavage *in vivo* (Fig. 3*A*). Analysis of proximal and distal segments of optic nerve following transection revealed fodrin cleavage in both the proximal and distal nerve segments (Fig. 5*B*), which is consistent with the appearance of a CRMP4 cleavage product in proximal and distal optic nerve (Fig. 2*E*). These data suggest that calpain could potentially cleave CRMP4 to generate tCRMP4 in response to axotomy. To directly test this, P4 to P7 DRG neurons were treated with recombinant calpain, which did indeed lead to the generation of a 55 kDa tCRMP4 cleavage fragment (Fig. 5*C*), supporting the idea that CRMP4 cleavage is a calpain-dependent process. We also asked whether the generation of the CRMP4 cleavage product could be efficiently blocked with a calpain inhibitor. DRG explants were grown on cell-filter inserts and axotomized by removing the cell bodies with a cell scraper. The axons on the underside of the filter were then collected and lysed for Western immunoblot. Severed axons revealed the presence of tCRMP4, and generation of this fragment was efficiently blocked with the calpain inhibitor ALLN (Fig. 5*D*). Because calpains can activate other enzymes that also contribute to degeneration such as caspases, we repeated the experiment in DRG explants isolated from *Caspase 3^−/−^* mice (Geden and Deshmukh, 2016). Similar to the wild-type DRG explants, axotomy-dependent cleavage of CRMP4 occurred in the axotomized *Caspase 3^−/−^* axons, and this was prevented by treatment with ALLN (Fig. 5*E*). These findings illustrate that axotomy is sufficient to drive the process of calpain-dependent CRMP4 cleavage.

Further, to address how CRMP4 may be cleaved proximal and distal to the lesion site in optic nerve while being restricted to the distal portion of the sciatic nerve, we asked whether CNS myelin-associated proteins affect CRMP4 cleavage. We found that the treatment of P4 to P7 DRG neurons with Nogo-22, an outgrowth inhibitory fragment of the CNS myelin-associated inhibitor Nogo-A, was sufficient to induce CRMP4 cleavage (Fig. 5*F*; Huebner et al., 2011), This is consistent with the finding that Nogo promotes calcium influx in neurons leading to the activation of calpain (Bandtlow and Löschinger, 1997). This raises the possibility that calpain activation and CRMP4 cleavage may occur both proximally and distally following optic nerve injury in response to Nogo, while it may be more spatially restricted in the PNS environment.

### CRMP4 cleavage fragments promote axon degeneration

**Figure 6.**
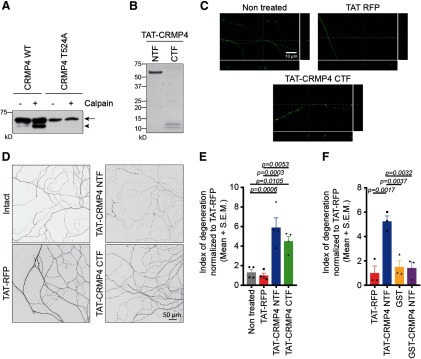
CRMP4 cleavage fragments promote axonal degeneration. ***A***, Western immunoblot analysis of WT-CRMP4S or CRMP4S-T524A in the presence or absence of calpain. Arrow, CRMP4S (65 kDa); solid arrowhead, tCRMP4 (55 kDa). ***B***, Coomassie staining of the TAT-CRMP4 peptides corresponding to the NTFs or CTFs. TAT-CRMP4-NTF and TAT-CRMP4-CTF produce peptides of ∼60 and 10–12 kDa, respectively. ***C***, Representative confocal pictures and orthogonal projections illustrating the integration of the TAT peptides (red) in the axons stained with Tuj1 (green). Scale bar, 10 μm. ***D***, ***E***, Representative pictures (***D***) and quantification of the index of degeneration (***E***) following treatment of the axons for 8 h with 2.5 μm TAT-CRMP4 peptides or with TAT-RFP as a control. The data in the graph represents the average fold change in the index of degeneration compared with TAT-RFP ± SEM (*n* = 4; one-way ANOVA, Tukey’s multiple-comparison test). Scale bar, 50 μm. ***F***, Quantification of the index of degeneration following treatment of the axons for 8 h with 2.5 μm TAT-or GST-CRMP4 NTF peptides, or with TAT-RFP and GST as controls. The data are shown as the average fold change in the index of degeneration compared with TAT-RFP ± SEM (*n* = 3; one-way ANOVA, Tukey’s multiple-comparison test).

To study the effects of CRMP4 cleavage fragments on neurons, we mapped the calpain cleavage site. Through sequential alanine substitutions of candidate residues in the C-terminal tail of CRMP4, we localized the specific cleavage site to residue T524 and demonstrated that a CRMP4 construct containing a T524A mutation was less sensitive to *in vitro* calpain digestion (Fig. 6*A*). We then generated N-terminal and C-terminal fragments of CRMP4 (CRMP4 NTF and CRMP4 CTF, respectively) as TAT peptides to mediate peptide internalization across the cell membrane (Fig. 6*B*,*C*; Torchilin, 2008; Khazaei et al., 2015). Dissociated wild-type E15-16 DRG neurons were grown in microfluidic devices, and the axons were treated for 8 h with TAT-CRMP4 peptides or with TAT-RFP as a control. Treatment with either TAT-CRMP4 NTF or CTF resulted in significant axonal degeneration, as characterized by the appearance of axonal swellings (Fig. 6*D*). The extent of degeneration was assessed by calculating the index of degeneration (Kilinc et al., 2011). This quantification revealed a significant effect of both CRMP4 fragments on axon degeneration compared with TAT-RFP (Fig. 6*E*; TAT-CRMP4 NTF, fold change of 5.19; TAT-CRMP4 CTF, fold change of 4.50), indicating that CRMP4 cleavage is sufficient to promote axonal degeneration. Combined treatment with TAT-CRMP4 NTF and CTF resulted in a similar significant effect on axon degeneration, indicating that the combination is not more potent *in vitro* (fold change of 6.89; *p* = 0.231). The addition of GST-CRMP4 NTF, which lacks the TAT membrane permeabilization sequence, failed to affect axon degeneration (Fig. 6*F*).

## Discussion

Following injury, peripheral neurons mount a regenerative response whereby a newly formed growth cone at the tip of the broken axon drives axon outgrowth, while the disconnected axon segments undergo Wallerian degeneration (Waller, 1850; Kamber et al., 2009; Ghosh-Roy et al., 2010; Bradke et al., 2012; Spira and Erez, 2013). Neuronal regeneration and degeneration are highly interdependent, and, as such, they share several signaling pathways (Bisby and Chen, 1990; Brown et al., 1992, 1994; Girouard et al., 2018). This is advantageous as both processes can be simultaneously targeted to promote an optimal regenerative response. Here, we characterized CRMP4 as a protein that contributes to both regeneration and degeneration following axotomy. CRMP4 plays a dual role in the neuronal response to PNS injury, as loss of CRMP4 impairs sensory axon regeneration by limiting axon regrowth and delaying Wallerian degeneration. These findings ascribe to a novel proregenerative role to CRMP4 in the PNS, contrasting to its role in the CNS, where it contributes to failed regeneration. Interestingly, these functions are very reminiscent of other proteins that regulate cytoskeletal dynamics and energy supply (Girouard et al., 2018). For example, SCG10/STMN2 accumulates in the regenerating axon segment of sensory neurons following peripheral axotomy to promote microtubule dynamics and axon regrowth (Shin et al., 2014). Conversely, it is degraded in the degenerating axon segment to accelerate axon fragmentation (Shin et al., 2012).

**Figure 7.**
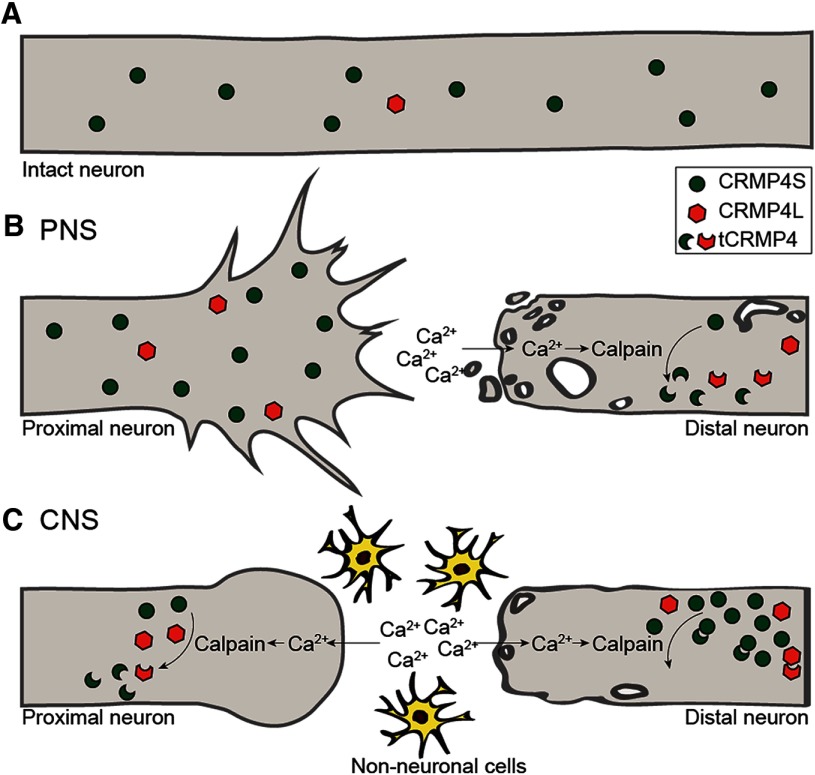
Schematic model illustrating the expression profile of CRMP4 following PNS and CNS injury. ***A***, In the intact sciatic and optic nerves, CRMP4S is strongly expressed, while levels of CRMP4L are very low. ***B***, In the sciatic nerve fibers located proximal to the injury site, the expression of CRMP4S is maintained, while CRMP4L is modestly upregulated. This expression pattern correlates with regeneration and contributes to axon extension. In the axon segments located distal to the injury site, the entry of calcium promotes the calpain-dependent cleavage of CRMP4, leading to the formation of tCRMP4 and the downregulation of full-length CRMP4, which promotes Wallerian degeneration. ***C***, In the optic nerve, CRMP4S is downregulated, while tCRMP4 is upregulated in the axons proximal to the lesion site, correlating with the formation of retraction bulbs and the inhibition of axon regeneration. In the axon fragments located distal to the injury site, the presence of tCRMP4 is accompanied by a sustained expression of CRMP4S.

Here, we find that, in the regenerating neurons, CRMP4 supports axon regrowth, while its calpain-mediated cleavage in the distal degenerating fibers facilitates Wallerian degeneration (Fig. 7*B*). It is notable that the relative ratio between full-length and truncated CRMP4 seems to be critical to its function (Fig. 7). In the injured PNS, full-length CRMP4 is exclusively expressed in the proximal segment correlating with axon regeneration. In the distal sciatic nerve, massive downregulation of full-length CRMP4 coinciding with the appearance of cleavage fragments correlates with Wallerian degeneration. In the optic nerve, CRMP4 expression in the proximal nerve segment mirrors the profile of the distal sciatic nerve, and this correlates with the formation of retraction bulbs and failed regenerative growth. While CRMP4 cleavage also occurs in the distal optic nerve, full-length CRMP4 is retained and may dimerize with truncated CRMP4 to buffer its neurotoxic activity. This is consistent with the idea that the overexpression of nonphosphorylated CRMP2 can protect axons from Wallerian degeneration (Wakatsuki et al., 2011).

Efficient neuronal regeneration requires the assembly of a functional growth cone that will drive axon extension (Bradke et al., 2012). This process is highly dependent on cytoskeletal remodeling, and, consequently, several cytoskeletal regulators are implicated in this process. Here, we find that CRMP4 contributes favorably to PNS regeneration, and this effect potentially stems from its regulatory functions toward the cytoskeleton. To support this hypothesis, hippocampal neurons lacking CRMP4 exhibit poorly elaborated growth cones with defects in their cytoskeletal organization (Khazaei et al., 2014; Tan et al., 2015). Thus, CRMP4 could potentially facilitate the cytoskeletal rearrangements required for the reformation of the growth cone, either via its direct interaction with actin and tubulin to promote their assembly into filaments or microtubules respectively, or via its interaction with CRMP2 (Rosslenbroich et al., 2005; Khazaei et al., 2014; Tan et al., 2015). Additionally, CRMP4L can interact with intersectin, an adaptor protein that regulates various cellular processes including endocytosis and exocytosis (Acheson et al., 1991; Yamabhai et al., 1998; Okamoto et al., 1999; Simpson et al., 1999; Hussain et al., 2001; Quinn et al., 2003). Thus, CRMP4L could play important roles in providing membrane and cell surface molecules important for axon regrowth. Consequently, CRMP4 could facilitate growth cone reformation in regenerating PNS axons, but also subsequent axon extension.

Conversely, loss of CRMP4 leads to a delay of Wallerian degeneration *in vitro.* The relative contribution of CRMP4 to growth of the proximal segment versus degeneration of the distal segment *in vivo* is an open question. In degenerating PNS axons, we observe the calpain-mediated cleavage of CRMP4 and concomitant downregulation of CRMP4S. Intriguingly, the expression pattern of CRMP4S and tCRMP4 observed in the sciatic nerve located distal to the injury site is very reminiscent of the CRMP2 expression profile following injury (Touma et al., 2007; Lin et al., 2011; Wakatsuki et al., 2011; Zhang et al., 2016). In the case of CRMP2, its inactivation either by phosphorylation or calpain-mediated cleavage impairs its microtubule stabilizing effects. Thus, it is likely that CRMP4 cleavage is mediating both gain-of-function toxicity in response to the accumulation of NTFs and CTFs as well as a loss-of-function phenotype (Touma et al., 2007; Lin et al., 2011; Wakatsuki et al., 2011; Zhang et al., 2016). Axon transport is disrupted in degenerating neurons, and overexpression of CRMP2 in axotomized cortical neurons rescues this defect and restores microtubule organization (Touma et al., 2007; Zhang et al., 2016). CRMP2 couples with tubulin heterodimers, proteins, and organelles to kinesin, allowing their anterograde transport (Kawano et al., 2005; Kimura et al., 2005; Arimura et al., 2009). As CRMP4 is also downregulated in degenerating neurons, this protein could also be involved in the maintenance of neuronal integrity, via the regulation of microtubule stabilization, axonal transport, or other functions that remain to be further explored. Additionally, calpain-mediated cleavage of CRMP4 leads to the generation of tCRMP4 fragments that are sufficient to trigger axonal degeneration in wild-type DRG neurons, which express endogenous levels of full-length CRMP4. CRMP4 and other CRMP family members assemble as hetero-tetramers to mediate their functions (Wang and Strittmatter, 1997). While it is likely that an excess of full-length CRMP4 expression would buffer against the deleterious effects of tCRMP4, it is also possible that tCRMP4 fragments could integrate in hetero-tetramers compromising their functionality. For example, CRMP2 and CRMP4 assemble as a complex that coordinates actin and microtubule dynamics in developing neurons (Tan et al., 2015). In addition to compromising cytoskeletal dynamics, cleavage fragments could affect CRMP-dependent roles in axon and mitochondrial transport, consequently promoting axonal fragmentation (Zhang et al., 2016).

The activity of cytoskeletal regulators is regulated by different kinases and upstream regulators in the regenerating and degenerating neurons (Girouard et al., 2018). Interestingly, these regulators also exhibit differential roles in the proximal and distal axon segments. MAP3K dual leucine zipper kinase 1 (DLK-1) functions through JNK and p38 MAPK to support transcriptional and translational events required for axon regeneration, while it regulates microtubules dynamics and energetics to favor degeneration of the distal axons (Miller et al., 2009; Watkins et al., 2013; Geden and Deshmukh, 2016). Additionally, nicotinamide mononucleotide acetyltransferase 1 (NMNAT1) is required for axonal integrity and, as such, in regenerating neurons, NMNAT1 preserves mitochondria to promote neuroprotection and support regeneration, whereas, in degenerating neurons, it limits degeneration by blocking NAD^+^ depletion (Chen et al., 2016; Sasaki et al., 2016). Thus, CRMP4, similarly to SCG10/STMN2 and CRMP2, could potentially act downstream of DLK-1 and NMNAT1, but this hypothesis will need to be further explored.

An interesting aspect of this study is the dichotomy between CRMP4 functions in the PNS compared with the CNS. CRMP4 plays an important role in the transduction of growth-inhibitory signals like MAIs and CSPGs (Alabed et al., 2007, 2010; Nagai et al., 2012, 2016). Following spinal cord injury, sensory neurons lacking CRMP4 exhibited enhanced growth in the dorsal horn, consistent with desensitization to MAIs and CSPGs (Nagai et al., 2016). Thus, the presence of myelin-associated inhibitory proteins and inhibitory glial scar components in the CNS, and their absence in the PNS likely explains the differential regulation of CRMP4 in the two systems. It is also apparent that, following spinal cord injury, CRMP4 is upregulated in the astrocytes surrounding the glial scar, whereas following peripheral nerve injury, CRMP4 is predominantly localized to damaged axons (Jang et al., 2010; Nagai et al., 2015). This diverging expression pattern suggests that, in the CNS, CRMP4 might possess additional functions in non-neuronal cells that would contribute to regeneration failure. While the restricted expression of CRMP4 in axons in the sciatic nerve suggests a neuron cell-autonomous role for CRMP4 in PNS regeneration, a neuron-specific CRMP4 deletion would be required to fully rule out additional roles for CRMP4 in inflammation or Schwann cell biology.

In conclusion, we characterized a dual role for the cytoskeletal regulator CRMP4 in the neuronal response to PNS injury, as it favors neuronal regeneration by promoting axonal regrowth and by facilitating Wallerian degeneration. Interestingly, these functions are very reminiscent of the growth-promoting effects of CRMP4 characterized in developing neurons, but differ from its growth-inhibitory action in the adult CNS. Understanding these differing functions is critical to the development of novel strategies to promote functional recovery following neuronal injuries.

## Synthesis

Reviewing Editor: Mike Fainzilber, Weizmann Institute of Science Decisions are customarily a result of the Reviewing Editor and the peer reviewers coming together and discussing their recommendations until a consensus is reached. When revisions are invited, a fact-based synthesis statement explaining their decision and outlining what is needed to prepare a revision will be listed below. The following reviewer(s) agreed to reveal their identity: NONE. Note: If this manuscript was transferred from JNeurosci and a decision was made to accept the manuscript without peer review, a brief statement to this effect will instead be what is listed below.

Both reviewers reviewed a previous version of this manuscript at J Neurosci. In this revised manuscript, the authors have improved the study and addressed most of the concerns raised in that previous review. The reviewers recommend proceeding with the revised manuscript after addressing the specific issues detailed below.

Some issues the authors must still address:

1) Quantification of the western blot analysis is required to conclude about CRMP4 dynamics in different regions. Although the authors state that the differences are clear and that the western blot images shown are representative of multiple replicates, quantification of the signal by normalization to the loading control is required. Specially considering they have the data to do so. This applies to all Western blot series performed in this study.

2) Degeneration is associated with several processes, including Schwann cell differentiation, axonal degeneration and macrophage infiltration. Delays in glial differentiation or macrophage infiltration can also lead to delayed regeneration. As they use a total KO animal, the lack of CRMP4 can impact other events that should be discussed. Even though they show that CRMP4 is expressed only in axons, a non-cell autonomous effect might still occur. The possibility of a contribution of Schwann cell reprogramming should be discussed.

## Author Response

The review comments are provided in their entirety with responses in Bold font. Synthesis of Reviews:

Both reviewers reviewed a previous version of this manuscript at J Neurosci. In this revised manuscript, the authors have improved the study and addressed most of the concerns raised in that previous review. The reviewers recommend proceeding with the revised manuscript after addressing the specific issues detailed below.

Some issues the authors must still address:

1) Quantification of the western blot analysis is required to conclude about CRMP4 dynamics in different regions. Although the authors state that the differences are clear and that the western blot images shown are representative of multiple replicates, quantification of the signal by normalization to the loading control is required. Specially considering they have the data to do so. This applies to all Western blot series performed in this study.

We have performed densitometry to quantify the CRMP4 Western Blotting data as requested. In the revised manuscript we have tracked changes to make it apparent where the results have been added.

2) Degeneration is associated with several processes, including Schwann cell differentiation, axonal degeneration and macrophage infiltration. Delays in glial differentiation or macrophage infiltration can also lead to delayed regeneration. As they use a total KO animal, the lack of CRMP4 can impact other events that should be discussed. Even though they show that CRMP4 is expressed only in axons, a non-cell autonomous effect might still occur. The possibility of a contribution of Schwann cell reprogramming should be discussed.

We have added a sentence to the discussion about potential non cell-autonomous roles of CRMP4.
